# Trends in computerized provider order entry: 20-year bibliometric overview

**DOI:** 10.3389/fdgth.2023.1217694

**Published:** 2023-07-11

**Authors:** Laura Gosselin, Romain Leguillon, Laetitia Rollin, Emeline Lejeune, Stéfan J. Darmoni, Julien Grosjean

**Affiliations:** ^1^ Department of Digital Health, Rouen University Hospital, Rouen, France; ^2^ Department of Pharmacy, Rouen University Hospital, Rouen, France; ^3^ Laboratoire D'Informatique Médicale et D'Ingénierie des Connaissances en e-Santé (LIMICS), U1142, INSERM, Sorbonne Université, Paris, France; ^4^ Institute of Occupational Medicine, Rouen University Hospital, Rouen, France

**Keywords:** bibliometric, bibliometric analysis, CPOE (computerized physician order entry), CPOE (computerized prescriber order entry), medical order entry systems

## Abstract

**Background:**

Drug-related problems (DRPs) can lead to serious health issues and have significant economic impacts on healthcare systems. One solution to address this issue is the use of computerized physician order entry systems (CPOE), which can help prevent DRPs by reducing the risk of medication errors.

**Objective:**

The purpose of this study is to provide an analysis on scientific production of the past 20 years in order to describe trends in academic publishing on CPOE and to identify the major topics as well as the predominant actors (journals, countries) involved in this field.

**Methods:**

A PubMed search was carried out to extract articles related to computerized provider order entry during the period January 1st 2003– December 31st 2022 using a specific query. Data were downloaded from PubMed in Extensible Markup Language (XML) and were processed through a dedicated parser.

**Results:**

A total of 2,946 articles were retrieved among 623 journals. One third of these articles were published in eight journals. Publications grew strongly from 2002 to 2006, with a dip in 2008 followed by an increase again in 2009. After 2009, there follows a decreasing until 2022.The most producing countries are the USA with 51.39% of the publication over the period by France (3.80%), and Canada (3.77%). About disciplines, the top 3 is: “medical informatics” (21.62% of articles), “pharmacy” (19.04%), and “pediatrics” (6.56%).

**Discussion:**

This study provides an overview of publication trends related to CPOE, which exhibited a significant increase in the first decade of the 21st century followed by a decline after 2009. Possible reasons for this decline include the emergence of digital health tools beyond CPOE, as well as healthcare professionals experiencing alert fatigue of the current system.

**Conclusion:**

Future research should focus on analyzing publication trends in the field of medical informatics and decision-making tools to identify other areas of interest that may have surpassed the development of CPOE.

## Background

Drug-related problems (DRPs) are a significant public health issue that affects patients worldwide. DRPs refer to any unwanted or harmful event associated with the use of medications, including adverse drug reactions, medication errors, and drug interactions. DRPs can result in hospitalization, disability, and even death, and they have a significant economic impact on healthcare systems (1, 2).

To address this issue, there are several solutions, including the use of computerized physician order entry (CPOE) systems to secure medication prescriptions (3). CPOE comes in two main types: hospital CPOE, which is used in healthcare institutions and limits drug choices to define lists, and ambulatory medicine CPOE. By using CPOE systems, healthcare professionals can help prevent DRPs by reducing the risk of medication errors, including incorrect dosages or medication interactions.

This study aims to assess the current trends in academic publishing on computerized physician order entry (CPOE) through proven bibliographic methods (4, 5). Specifically, it involves conducting a mapping review to identify the key contributors to the field (e.g., journals, countries), the most advanced medical disciplines on the subject, the prevalent themes, and the evolution of published articles since the inception of CPOE.

To our knowledge, no bibliometric analysis of CPOE has been carried out. It is important to conduct this research to map and analyze publications related to CPOE. This analysis can help identify weaknesses and improve CPOE research. Other works have consisted in drawing up a history of CPOEs but without a macroscopic vision or in a way that is too descriptive of the very principles of the discipline (6, 7). More methodologically similar studies have appeared but only on topics such as drug errors and adverse drug reactions in 2019 (8).

## Methods

### Bibliographic research

This study utilized the MEDLINE bibliographic database, which contains a vast collection of scientific articles from the biomedical field spanning several decades. The database's search engine, PubMed, enables the use of MeSH thesaurus keywords for precise indexing of articles, and it also permits searching for terms in unstructured fields such as article titles and abstracts. Additionally, the Boolean-compatible syntax, featuring the use of operators such as AND, OR, and NOT, provides exceptional flexibility for conducting queries.

The following query was constructed with the help of a medical librarian (EL) to select articles indexed with the keywords “Computerized Physician Order Entry” (MeSH identifier D050316) between 2003 and 2022 (a period of 20 completed years). To increase recall, the terms “CPOE”, “Computerized Physician Order Entry”, “computerized physician order entry system”, “computerized Provider Order Entry”, “Computerized Provider Order Entry System”, “prescription tool”, “prescription support tool”, and “medication alert system” were searched in the title. In order to minimize noise (false positives), some terms were excluded such as “laboratory test” or “laboratory”. The final equation is as follows:

“medical order entry systems"[MH] OR “medical order entry system*"[TW] OR CPOE[TW] OR “Computerized Physician Order Entry"[TW] OR “computerized physician order entry system"[TW] OR “computerized Provider Order Entry"[TW] OR “Computerized Provider Order Entry System"[TW] OR “prescription tool*"[TW] OR “prescription support tool*"[TW] OR “medication alert system*"[TW] NOT “laboratory test*"[TW] NOT “laboratory"[TW]

### Extraction and processing of data

The bibliographic references retrieved from PubMed through the query are in XML (eXtensible Markup Language) format. A specialized program is used to extract relevant data from selected metadata, including the year of publication, author names and affiliations, journal, MeSH keywords, publication type, and language.

Two additional metadata were automatically added using specialized algorithms. First, the country of publication was inferred from the affiliation of the first author, which is typically the most informed and representative of the article's content. A computer program utilizing the Google Maps API and a dedicated database was used to identify the country from the author's email domain or location. Second, medical specialties were assigned to each reference based on the MeSH keywords using a categorization algorithm developed previously (9). The algorithm leverages the MeSH hierarchy and relations defined by domain experts to associate each keyword with one or more medical specialties (10). We conducted a human verification of a random sample of 100 articles related to CPOE (evaluation, design, and use of CPOE) by two individuals (LG and JG). This verification involved assessing the titles and abstracts of the articles to ensure that the retrieved results are relevant to CPOE.

Further information on the yearly publication count in MEDLINE was directly obtained from PubMed. Moreover, the total number of publications from these journals during the study period was compiled in the data table sourced from PubMed. Population data, including population size and Gross Domestic Product (GDP) per capita by country, were sourced from the World Population Review (http://worldpopulationreview.com) and the World Bank (https://data.worldbank.org/indicator).

Bradford's law was used to rank the journals. Bradford's law is also known as Bradford's law of scattering or the Bradford distribution, as it describes how the articles on a particular subject are scattered throughout the mass of periodicals (11).

The data were compiled into a table, enabling the creation of sub-tables and corresponding graphs.

### Analyse of datas

The different data were extracted from our tool in the form of an Excel® spreadsheet. The columns ID MeSH, type of publication, N, and proportion of articles (%) were extracted directly.

## Results

**Figure 1:**
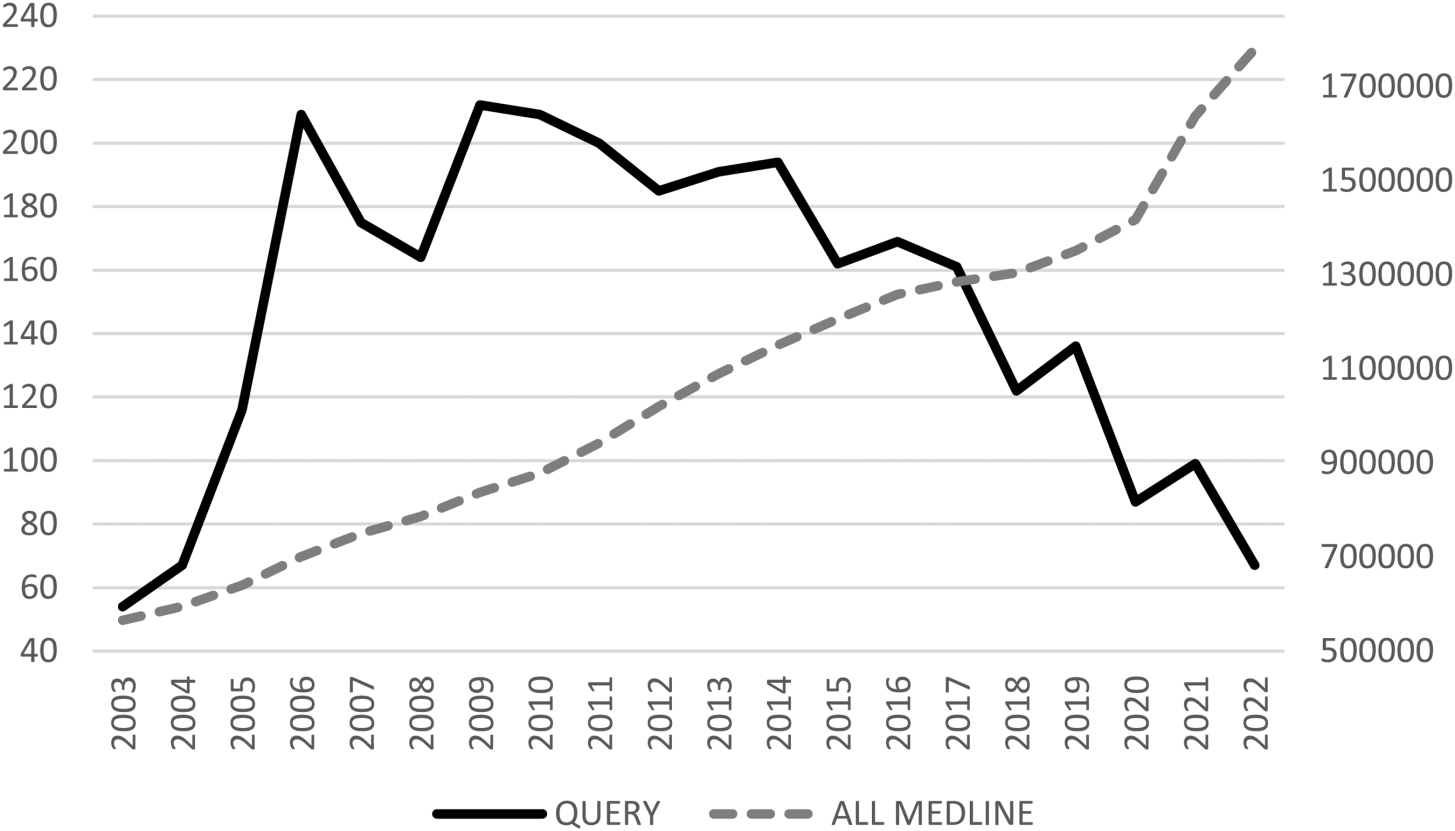
Evolution of the number of articles per year for MEDLINE and for the query.

PubMed query yielded a total of 2,946 articles. Out of the 100 articles in the sample, 100% were found to be related to CPOE. As shown in Figure 1, there was significant growth from 2002 to 2006, followed by a decline in 2008 and subsequent increase in 2009. However, from 2009 onwards, there was a downward trend until 2022, with the highest number of articles (212 references) published in 2009 and only 67 articles published in 2022. The average annual increase in articles on the subject was 1.27% [−0.36; 23.00].

### Journals

Out of the 623 journals that published at least one article identified by the query, only five published over 100 articles, and 13 published more than 30 articles. Bradford's law was used to rank the journals, with eight journals publishing one third of the identified articles (1.8% of the journals), 59 journals publishing the second third (9.47% of the journals), and the rest of the journals publishing the final third, representing the vast majority (89.24%). Among the 13 journals that published more than 30 articles, 7 (53.85%) were specialized in the field of medical informatics. Table 1 shows that none of the main journals identified had a proportion of articles exceeding 10%. The Journal of the American Medical Informatics Association (JAMIA) had the highest proportion of articles at 7.71%.

**Table 1:** Top 50 journals that published the articles identified by the query.

Journal	N	Proportion (%)
Journal of the American Medical Informatics Association: JAMIA	227	7.71%
Studies in Health technology and Informatics	205	6.96%
Annual Symposium proceedings. AMIA Symposium	152	5.16%
American Journal of Health-system Pharmacy: AJHP: official journal of the American Society of Health-System Pharmacists	129	4.38%
International Journal of Medical Informatics	117	3.97%
Healthcare Informatics: the business magazine for information and communication systems	70	2.38%
Applied Clinical Informatics	64	2.17%
Pediatrics	44	1.49%
Journal of Healthcare Information Management: JHIM	40	1.36%
Healthcare Quarterly (Toronto, Ont.)	36	1.22%
Journal of the American College of Radiology: JACR	34	1.15%
Joint Commission Journal on Quality and Patient Safety	31	1.05%
BMC Medical Informatics and Decision Making	31	1.05%
Modern Healthcare	29	0.98%
Journal of Medical Systems	29	0.98%
Health Management Technology	25	0.85%
Journal of General Internal Medicine	25	0.85%
International Journal of Clinical Pharmacy	24	0.81%
Journal of Hospital Medicine	23	0.78%
Hospitals & Health Networks	22	0.75%
Methods of Information in Medicine	21	0.71%
Yearbook of Medical Informatics	21	0.71%
BMJ Quality & Safety	20	0.68%
Archives of Internal Medicine	19	0.64%
Computers, Informatics, Nursing: CIN	19	0.64%
Quality & Safety in Health Care	18	0.61%
Health Affairs (Project Hope)	18	0.61%
JAMA	17	0.58%
Drug Safety	17	0.58%
PloS One	17	0.58%
Transfusion	16	0.54%
Healthcare Benchmarks and Quality Improvement	16	0.54%
Journal of Biomedical Informatics	16	0.54%
Annals of Emergency Medicine	15	0.51%
Journal of the American Geriatrics Society	14	0.48%
Health Data Management	14	0.48%
The Annals of Pharmacotherapy	14	0.48%
The American Journal of Medicine	13	0.44%
Health Informatics Journal	12	0.41%
BMJ Open	12	0.41%
Journal of Patient Safety	12	0.41%
The American Journal of Emergency Medicine	12	0.41%
Nursing Management	12	0.41%
The American Journal of Managed Care	12	0.41%
European Journal of Clinical Pharmacology	12	0.41%
American Journal of Medical Quality: The Official Journal of the American College of Medical Quality	11	0.37%
AJR. American Journal of Roentgenology	11	0.37%
International Journal for Quality in Health Care: Journal of the International Society for Quality in Health Care	11	0.37%
Journal of Oncology Practice	11	0.37%
JMIR Medical Informatics	10	0.34%

### Languages

Most of the articles were written in English (96.9%), with Spanish and French, as well as German and Danish, accounting for 0.8% to 0.4% of the articles, respectively (Table 2). In MEDLINE, English was the predominant language at 97.89%, followed by Chinese at 0.62% and German at 0.52%. Nowadays, English is the predominant language for writing scientific articles, so the trend is quite normal.

**Table 2:** Language distribution of the articles identified by the query.

Language code	N	Proportion of articles (%)
en (english)	2,856	96,95%
fr (french)	22	0,75%
es (spanish)	22	0,75%
de (deutch)	19	0,64%
da (danish)	9	0,31%
no (norwegian)	5	0,17%
se (sweden)	4	0,14%
pt (portuguese)	3	0,10%
ja (japenese)	2	0,07%
it (italian)	2	0,07%
ko (korean)	1	0,03%
he (hebrew)	1	0,03%

### Countries

Out of the 2,946 articles, 2,516 (85.4%) had a first author affiliation that identified a country. Table 3 provides a breakdown of the number of articles and ratios according to population size and GDP per capita (PPP, in international dollars). The United States of America led with 51.39% of the articles (among countries identified), followed by France (3.80%), Canada (3.77%), Australia (3.36%), and the United Kingdom (3.12%). The top 5 countries with the highest number of articles per 100,000 inhabitants were Australia (1.1), Switzerland (0.59), the United States of America (0.46), the Netherlands (0.45), and Denmark (0.36). Meanwhile, the top 5 countries with the highest number of articles per GDP (in PPP per 1,000 international dollars) were the United States of America (23.95), France (2.38), Canada (2.38), Australia (1.86), and the United Kingdom (1.66).

**Table 3:** Top 25 countries associated with the items identified by the query.

Countries	Rank	Number of articles	Proportion of articles (%)	Number of inhabitants	Number of articles per 100,000 inhabitants	GDP PPA ($)	Number of articles per GDP PPA KGK ($)
United States of America	1	1,514	51.39%	331 449 281	0.46	63 206.52	23.95
France	2	112	3.80%	66 043 511	0.17	46 991.18	2.38
Canada	3	111	3.77%	37 742 154	0.29	46 572.14	2.38
Australia	4	99	3.36%	9 006 398	1.10	53 316.89	1.86
United Kingdom	5	92	3.12%	67 886 011	0.14	55 300.00	1.66
Netherlands	6	77	2.61%	17 134 872	0.45	59,266.91	1.30
Spain	7	55	1.87%	46 754 778	0.12	37 756.35	1.46
Switzerland	8	51	1.73%	8 654 622	0.59	71 745.30	0.71
Taiwan	9	38	1.29%	23 816 775	0.16	55 720.00	0.68
Germany	10	37	1.26%	83 783 942	0.04	54 844.55	0.67
South Korea	11	35	1.19%	51 269 185	0.07	45 225.84	0.77
Saudi Arabia	12	27	0.92%	34 813 871	0.08	46 759.66	0.58
Japan	13	22	0.75%	126 239 461	0.02	42 390.38	0.52
Denmark	14	21	0.71%	5 792 202	0.36	60 229.91	0.35
Sweden	15	21	0.71%	10 099 265	0.21	55 037.72	0.38
Austria	16	20	0.68%	9 006 398	0.22	55 685.97	0.36
Italy	17	17	0.58%	60 628 826	0.03	41 902.08	0.41
China	18	16	0.54%	1 425 893 465	0.00	17 210.76	0.93
Iran	19	15	0.51%	83 992 949	0.02	13 338.01	1.12
Argentina	20	14	0.48%	45 195 774	0.03	20 770.73	0.67
Belgium	21	14	0.48%	11 589 623	0.12	53 088.97	0.26
Israel	22	12	0.41%	8 655 535	0.14	39 489.28	0.30
Ireland	23	9	0.31%	4 937 786	0.18	93 350.09	0.10
Brazil	23	9	0.31%	217 374 417	0.00	14 835.41	0.61
Pakistan	24	7	0.24%	220 892 340	0.00	4 812.89	1.45

### Types of publication

To filter out uninformative types such as grant-related publication types like “non-U.S. government research grant”, “NIH extramural research grant”, “U.S. government PHS research grant”, and “NA”, we retained 36 relevant publication types. The top 15 publication types account for almost all articles (see Table 4). These include journal article (93.73% of the relevant publication types), literature reviews (5.23%), comparative studies (3.36%), evaluation studies (2.47%), and commentaries (2.16%). The data in Table 4 are raw, and an article can have multiple types of publication.

**Table 4:** Top 15 publication types (MeSH) of articles identified by the query.

ID MeSH	Type of publication	N	Proportion of articles (%)
D016428	Journal Article	2,761	93.72
D016454	Review	252	8.55
D003160	Comparative Study	162	5.50
D023362	Evaluation Study	119	4.04
D016420	Comment	104	3.53
D016422	Letter	93	3.16
D016448	Multicenter Study	82	2.78
D016449	Randomized Controlled Trial	59	2.00
D004740	English Abstract	56	1.90
D016421	Editorial	53	1.80
D064888	Observational Study	53	1.80
D016433	News	25	0.85
D017418	Meta-analysis	21	0.71
D023361	Validation Study	20	0.68
D016430	Clinical Trial	18	0.61

### MeSH terms

In total, 2,946 articles were identified in the study, which were indexed with 1,788 unique MeSH keywords. Check tags were excluded from the analysis as they did not provide any useful information. Table 5 presents the top 50 most frequently occurring keywords. The most common keywords were «Medical order entry systems» (68.53%), «Medication errors» (28.89%), «clinical decision support systems» (20.88%), and «United States» (16.63%).

**Table 5:** Top 50 MeSH keywords that index the identified articles.

ID MeSH	MeSH terms	N	Proportion of articles (%)
D050316	Medical Order Entry Systems	2,019	68.53
D008508	Medication Errors	851	28.89
D020000	Clinical Decision Support Systems	615	20.88
D014481	United States	490	16.63
D016347	Computerized Medical Record Systems	360	12.22
D057286	Electronic Medical Records	357	12.12
D008510	Hospital-Based Drug Dispensing And Distribution Systems	316	10.73
D011307	Medication Orders	251	8.52
D000368	Elderly Subject	250	8.49
D012189	Retrospective Studies	222	7.54
D010818	Types Of Physician Practices	212	7.20
D055695	Electronic Prescribing	208	7.06
D014584	User Interface	205	6.96
D004360	Computer-Assisted Drug Therapy	204	6.92
D017751	Safety Management	196	6.65
D001291	Attitude Of Health Care Personnel	194	6.59
D004059	Diffusion Of Innovations	188	6.38
D016303	Clinical Pharmacy Information Systems	179	6.08
D006751	Hospital Information Systems	172	5.84
D010607	Hospital Pharmacy	171	5.80
D010820	Physicians	170	5.77
D019300	Medical Errors	140	4.75
D064420	Adverse Drug Reactions	137	4.65
D000900	Antibacterials	136	4.62
D061214	Patient Safety	128	4.34
D004347	Drug Interactions	126	4.28
D017598	Efficacy And Effectiveness	125	4.24
D011446	Prospective Studies	125	4.24
D001292	Computer Skills	124	4.21
D010595	Pharmacists	119	4.04
D012984	Software	117	3.97
D013997	Time Factors	115	3.90
D004364	Pharmaceutical Preparations	110	3.73
D011785	Quality Assurance In Health Care	110	3.73
D000369	Elderly Subject 80 Years Or Older	107	3.63
D009936	Organizational Innovation	103	3.50
D017010	Memory Aids	102	3.46
D019983	Adherence To Guidelines	102	3.46
D000046	Teaching Hospitals	97	3.29
D008490	Medical Informatics	95	3.22
D017410	Good Clinical Practice Guidelines As A Topic	93	3.16
D018511	Systems Integration	89	3.02
D019982	Case Studies Of Health Care Organizations	88	2.99
D011787	Quality Of Health Care	87	2.95
D004636	Hospital Emergency Department	86	2.92
D006761	Hospitals	86	2.92
D000925	Anticoagulants	82	2.78
D000970	Antineoplastics	80	2.72
D007362	Intensive Care Units	78	2.65
D006785	Teaching Hospitals	78	2.65

### Medical specialties

The categorization algorithm is grounded on MeSH keywords to infer broad categories, particularly in the field of health, medicine, or paramedical disciplines. As a result, a total of 85 disciplines were identified among the articles (representing 60.28% out of 141 disciplines in total) as presented in Table 6. The top 5 disciplines include “medical informatics” accounting for 21.62% of the articles, followed by “pharmacy” at 19.04%, “pediatrics” at 6.56%, “medicine” at 5.45%, and “geriatrics” at 4.49%.

**Table 6:** Health disciplines calculated via the categorization algorithm from the MeSH keywords indexing the identified articles.

Medical specialty	N	Proportion of medical specialties (%)
Medical Informatics	41,996	21,62%
Pharmacy	36,975	19,04%
Pediatrics	12,740	6,56%
Medicines	10,583	5,45%
Geriatrics	8,728	4,49%
Nursing	7,993	4,12%
Evidence-Based Medicine	7,270	3,74%
Medical Imaging	5,383	2,77%
Ambulatory Medicine	4,967	2,56%
Medical And Surgical Resuscitation	4,410	2,27%
Oncology	4,069	2,09%
Risk Management	3,759	1,94%
Bacteriology	3,610	1,86%
Pharmacology	3,513	1,81%
Diagnosis	3,042	1,57%
Medical Education	2,687	1,38%
Occupational Medicine	2,646	1,36%
Neonatology	2,434	1,25%
Therapeutics	2,251	1,16%
Medical Devices	1,757	0,90%

## Discussion

The main results of this study provide us with trends in publications related to CPOE. Indeed, the first decade of the 21st century was marked by an increase in publications each year, which is correlated with the implementation of health information systems. Today, whether for hospital or primary care, CPOE systems are widely implemented in the daily practices of different healthcare professionals.

However, after 2009, there has been a clear decrease in the number of publications regarding CPOE, despite strong pressure from public authorities to continue digitizing healthcare systems. One possible line of thought that can be suggested is that the digitization of health tools is not only through CPOE (artificial intelligence, decision support, etc.) but also through a certain exhaustion of professionals towards CPOE. On the other hand, CPOE are now totally integrated in most hospital information systems and in software in primary care, as well as in some health information systems (at a national level, e.g., Israel, Denmark, Taiwan, Singapore).

Regarding Figure 1, the curve of our query decreases from 2009 while the trend of articles published on Medline only increases. Over the course of the past 20 years, the field of Computerized Physician Order Entry (CPOE) has witnessed a significant evolution, transitioning from an innovative technology to a mature one. This shift can be attributed to several factors.

Initially, when CPOE was introduced, it was considered a groundbreaking technology with the potential to revolutionize healthcare systems. Its implementation was met with enthusiasm and high expectations for improving patient safety, reducing medication errors, and enhancing overall workflow efficiency. Researchers and practitioners were eager to explore its capabilities and document its impact through academic publications.

As CPOE became more widely adopted and integrated into healthcare organizations, the initial wave of excitement and novelty subsided. With increased implementation, researchers began to shift their focus from simply exploring the technology's benefits to evaluating its real-world effectiveness and identifying areas for improvement. This transition from exploration to evaluation is characteristic of the maturation process.

Moreover, as CPOE became more commonplace, it started to be considered a standard practice rather than an innovative solution. Healthcare organizations began to expect its implementation as part of their electronic health record (EHR) systems. Consequently, the emphasis shifted from proving the concept to refining and optimizing the existing implementations. This shift in focus led to a decline in the number of publications specifically addressing the “acceptance” and “implementation” of CPOE.

Concerning the journals of publication and the medical specialties most found, medical informatics and digital health is predominant (e.g., JAMIA). Finally, few other specialties are interested or at least publish on this subject, except for pharmacy (concerned with drug prescribing and prescription analysis (e.g., AJHP), and care units such as pediatrics and geriatrics in which drug errors are more likely to occur.

Despite the ongoing issues of medication errors, the first impression is that researchers have come full circle about CPOE. This topic is becoming obsolete despite the fashion for artificial intelligence (AI) and all the possibilities for improving these CPOE coupled with AI.

Some countries, such as Canada, the United Kingdom (12) and Australia (13), are known to have a highly developed clinical pharmacy activity. This activity is based on good IT tools and good CPOE. Along with the United States and France, they are the countries that publish the most (in terms of the number of articles per 100,000 inhabitants).

It should be emphasized that CPOE are programmed with pre-established rules, which is called symbolic artificial intelligence. Today, there is shift towards digital artificial intelligence that is taking over thanks to all the health data that has been digitized. These two types of artificial intelligence deserve to be brought together in order to build a more efficient and personalized CPOE based on the prescriber and their prescribing habits.

Several studies highlight that the alert system of CPOE leads to a certain weariness of healthcare professionals who eventually stop using these tools in their entirety (14, 15).

Finally, another of our hypotheses concerning the decline in publications in this field is the change in research and economic models. Indeed, the research and publication field are mainly public, while it is the private software industry that creates the CPOE. The research and development sector of private industries is in sharp decline, and start-ups that create upstream software models are bought and developed by large software companies, which may tend to a decrease in scientific publication.

CPOE systems help reduce medication prescribing errors, such as dosage errors, potential drug interactions, and drug allergies. They facilitate communication among different healthcare professionals involved in patient care, thereby reducing communication errors and misunderstandings. Additionally, they can provide alerts and clinical guidance based on best practices and drug information, assisting prescribers in making more informed decisions and avoiding errors. Lastly, CPOE systems allow for tracking of medical orders, thereby facilitating monitoring of administered medications, detection of potential errors, and evaluation of therapeutic regimens. For future research, researchers could build upon the findings of our bibliometric analysis to design more effective and user-friendly CPOE systems. Indeed, alert fatigue remains a major usability barrier for CPOE systems, as described earlier. Additionally, some countries may consider investing in research on CPOE to mitigate medication prescribing errors.

It should be noted that our study is limited by the fact that it is based solely on Pubmed and not on all other search engines that may contain articles on CPOE, which may underestimate the number of publications in the field. Secondly, the tool used to perform this query is based on the categories and indexations of PubMed, which may not be indicated or may not appear in some articles. Then, one limitation of the article is the absence of subsequent examination of the included publications. As a result, our observation of a decrease in original articles or editorials/perspectives/letters published after 2009 lacks precision. This type of study aims to provide a comprehensive overview of the subject and pave the way for further investigations. One perspective that could be undertaken is to study trends in publication in the literature in the field of medical informatics and prescription and/or decision support tools. This would allow us to know if there is another area of interest that has taken over the development of CPOE.

## Conclusion

In conclusion, we can observe that research concerning CPOE is uneven across countries. The disciplines with the highest publication rates are those with the most interest in CPOE, such as pharmacy and pediatrics. Possible reasons for the decline of publication trends related to CPOE after 2009 include the emergence of digital health tools beyond CPOE, as well as healthcare professionals experiencing fatigue with the alert functions of the current system. Moreover, changes in the research and development sector may have contributed to the decrease in scientific publications. Future research should focus on analyzing publication trends in the field of medical informatics and decision-making tools to identify other areas of interest that may have surpassed the development of CPOE.
